# The developmental relation between aggressive behaviour and prosocial behaviour: A 5-year longitudinal study

**DOI:** 10.1186/s40359-015-0073-4

**Published:** 2015-05-14

**Authors:** Ingrid Obsuth, Manuel P Eisner, Tina Malti, Denis Ribeaud

**Affiliations:** Institute of Criminology, University of Cambridge, Sidgwick Site, Cambridge, CB3 9DA UK; Department of Psychology, University of Toronto, 3359 Mississauga Rd. N., Mississauga, ON L5L 1C6 Canada; Criminological Research Unit, Chair of Sociology, Swiss Federal Institute of Technology (ETH Zurich), WEP H18, Weinbergstrasse 109, 8092 Zürich, Switzerland

**Keywords:** Aggressive behaviour, Prosocial behaviour, Peer difficulties, Longitudinal study, Childhood

## Abstract

**Background:**

Past research has shown links between both children’s aggressive behaviour and a lack of prosocial behaviour to later maladaptation. Both types of behaviours have also been identified as crucial in children’s social and emotional development and later (mal)adaptation. However, little is known about the way they predict each other over time.

**Methods:**

We utilised a large, ethnically diverse, longitudinal population sample of girls and boys (N = 1,334) to examine the bidirectional cross-lagged links between aggressive and prosocial domains of behaviour from age seven to eleven. Teacher, parent and child self-reports were utilised to assess aggressive behaviour and prosocial behaviour.

**Results:**

The results revealed that aggressive behaviour measured one year predicted decreases in prosocial behaviour in the following year. Conversely, prosocial behaviour did not predict changes in aggressive behaviour in the subsequent year. Furthermore, peer difficulties were examined and found to be an important mediator of the link between aggressive and prosocial behaviour. Specifically, peer difficulties mediated the links between aggressive behaviour and prosocial behaviour one year later, particularly during the first three years of school attendance.

**Conclusions:**

Implications of the findings for the design of intervention strategies to reduce children’s aggressive behaviour are discussed.

## Background

Numerous studies have shown that aggressive behaviour and prosocial behaviour are negatively correlated concurrently at different stages of development (e.g., Eivers et al. 2012; Krahé and Möller 2011). Yet, few studies examined the possible impact of these behaviours on each other over time and very little is known about the developmental processes which may facilitate the link between them. The present study sets out to fill these important research gaps.

Aggressive behaviour has been defined as any behaviour directed towards another person that is carried out with the proximate intent to cause physical or psychological harm (Krahé 2013). Prosocial behaviour is social behaviour that benefits another person (Eisenberg et al. 2015). Early developmental research conceptualised the relation between aggressive and prosocial behaviour as two poles of the same behavioural construct, which would suggest that the two constructs co-vary at any moment in time. When Wispe (1972) first introduced the term ‘prosocial behaviour’ over four decades ago, she defined it as the opposite of ‘antisocial behaviour’, including aggressive behaviour. Consistently, some researchers (e.g., Eron and Huesmann 1984; Wiegman and van Schie 1998) argue that the respective underlying variables represent opposite ends of one broader construct based on evidence that prosocial behaviour is positively and aggressive behaviour is negatively related to common third variables, such as empathy (e.g., Eisenberg and Miller 1987). However, others have established that aggressive versus prosocial behaviour are two related but distinct behavioural constructs (e.g., Caprara et al. 2006) and find that each contributes unique variance in relation to explaining later negative and positive developmental outcomes.

Arguably, the two behaviours are conceptually related constructs, and numerous developmental theories, such as Bowlby’s (1980) attachment theory and socialisation theories (e.g., Hastings et al. 2007) have elaborated that both behaviours result from similar causal mechanisms. For example, by experiencing their parents as empathic and trustful individuals towards them and to each other, children learn how to be other-oriented and prosocial (Eisenberg et al. 2015). On the other hand, in situations where parents do not show empathy and trust in them, children may respond with aggressive behaviour.

Despite this conceptual notion, researchers have only relatively recently started to study both behaviours simultaneously using longitudinal designs (e.g., Caprara et al. 2001; Eivers et al. 2010) in order to understand their developmental links. The majority of these studies have examined the individual correlates as well as stability and change of these two behaviours, but not their impact on each other over time. For example, in a longitudinal study of 800 participants at ages 8, 19 and 30, Eron and Huesmann (1984) found that prosocial behaviour was negatively related to aggressive behaviour consistently at each point in time. Caprara et al. (2006), on the other hand, found that while they were related, the degree of the concurrent relation between prosocial and aggressive behaviour varied depending on the age of the child over a six year period (from age 7 to 13) and the informant; these researchers thus argued against considering them as mere opposite ends of a single construct. Similarly, Kokko et al. (2006) investigated the link between the developmental trajectories of physically aggressive and prosocial behaviour in a large male sample assessed at ages 6, 10, 11 and 12. They identified three trajectories of aggression – low, moderate and high declining trajectories; and contrary to expectations, only two trajectories of prosociality – low and moderate declining. Boys in the low aggression trajectory group were evenly distributed in the low and moderate prosocial trajectory groups. However, the majority of boys in the moderate aggression trajectory group (63%) and high aggression trajectory group (79%) followed the low prosociality trajectory. While these findings suggest an inverse relation between aggressive and prosocial trajectories, this study did not elucidate how the behaviours may be relating to each other over time. It also remains unclear whether these findings generalise to a normative sample of boys and girls. Furthermore, when examining the links between the aggressive and prosocial behaviour trajectories, in the same study Kokko et al. (2006) found that while physical aggression predicted both school dropout and physical violence at age 17, prosocial behaviour did not serve as a protective factor for the same behaviours. This pattern of findings is contrary to those presented in an earlier study by Crick (1996), who found that prosocial behaviour was uniquely related to future peer acceptance and peer rejection when accounting for aggressive behaviour. These inconsistent findings, along with the overall paucity of research in this area, highlight the importance of further examining the longitudinal directional links between aggressive and prosocial behaviour. Although there is some limited empirical evidence supporting a (negative) association between the two behaviours over time, the cross-lagged bidirectional relation between them has not been examined.

Insight into the dynamic relations between aggressive behaviour and prosocial behaviour is of importance for both conceptual and practical reasons. Conceptually, both behaviours are morally relevant, since they both concern the compliance with or infringement of moral norms, such as concern for the welfare of others, justice and fairness, or the omission of physical and psychological harm (Malti and Krettenauer 2013; Eisner and Malti 2015). Practically, understanding whether one can expect that desirable change in one type of behaviour is linked to subsequent change in the other type, may have implications for existing intervention strategies as well as for the design of new programmes. For example, if increases in prosocial behaviour result in decreases in aggressive behaviour, interventions may focus on increasing the former to achieve results on the latter. However, if this direct link is not present, interventions would need to incorporate strategies to achieve decreases in aggression through other mechanisms, such as peer rejection.

Three possible developmental links are plausible between these two behaviours: First, only prosocial behaviour predicts future aggressive behaviour. Second, only aggressive behaviour predicts future prosocial behaviour. Third, aggressive and prosocial behaviours reciprocally predict each other over time. Each of these possible links will be further discussed below.

### Prosocial behaviour predicts subsequent aggressive behaviour

Some developmental scientists have argued that levels of prosocial behaviour may be inversely linked to the risk of subsequent aggressive behaviour (e.g., Chen et al. 2000; Pursell et al. 2008). For example, peer reports of prosocial behaviour at age 12 were negatively related to teacher reports of behaviour problems at age 14 (Chen et al. 2000). Such a pathway may result from peer dynamics in that children with low prosocial behaviour can be expected to be rejected by socially competent friends (e.g., Ladd 1999; Vitaro et al. 1990), which in turn increases their risk of aggressive behaviours (e.g., Dodge et al. 2013; Lansford et al. 2010; Ostrov 2010).

Prevention and intervention programmes for children at risk for aggressive behaviour problems frequently target the enhancement of prosocial skills with the goal to increase prosocial behaviours (Sheridan et al. 2011) and decrease aggression (Conduct Problems Prevention Research Group, 2010). Meta-analytic evidence suggests positive effects of life skills and social-emotional learning programmes on aggressive problem behaviour (e.g., Durlak et al. 2011; Malti T, Chaparro MP, Zuffianò A, & Colasante T. School-based interventions to promote empathy in children and adolescents: A developmental analysis, Submitted). Recently, researchers have begun to examine the mechanisms of change (i.e., mediating variables) related to reductions in aggression. One meta-analysis (Dymnicki et al. 2011) identified social skills, social-cognitive processes, and classroom characteristics as mechanisms linked to small but significant reductions in overt aggression following universal school-based violence prevention programmes. However, it remains unknown whether the reductions in aggression may indeed be mediated by increases in prosocial behaviour.

### Aggressive behaviour predicts subsequent prosocial behaviour

Other developmental scientists have argued that aggressive behaviour may be linked to subsequent reductions in prosocial behaviour, particularly if children form friendships with aggressive peers (e.g., Bowker et al. 2007). Empirical findings suggest that aggressive children tend to form friendships with each other (Dishion and Tipsord 2011; Logis et al. 2013), they lose their social reputation, and experience peer rejection. When they attack and inflict harm on others, aggressive children may be seen as a threat to both victims and bystanders, who may therefore avoid interactions with them. In this way, children who engage in aggressive behaviour may isolate themselves from and/or become isolated by their socially competent peers from whom they could learn to engage in prosocial behaviours. In addition, aggressive behaviour, especially when it is part of a sustained pattern of conduct problems, is likely to reinforce social information-processing biases (Arsenio and Lemerise 2004). Hence, children who engage in aggressive behaviour may subsequently not perceive prosocial behaviours as response options and/or they may not evaluate them as strategies that are associated with internal or external gratifications.

### Aggressive behaviour and prosocial behaviour reciprocally relate to each other over time

The third possibility is that aggressive behaviour and prosocial behaviour reciprocally relate to or predict each other over time. Zimmer-Gembeck et al. (2005) examined but did not find reciprocal links between prosocial behaviour and relational or physical aggression and vice versa between Grades 3 and 6. They did, however, find that social preference, a measure of likability and acceptance by peers, predicted both aggressive behaviours as well as prosocial behaviour three years later. To our knowledge only one study thus far has examined the possibility of reciprocal links between these two behaviours at more than only two time points and across a longer period of time. Specifically, Chen et al. (2010), tested the cross-lagged reciprocal relations between aggressive behaviour, academic achievement and social competence, a construct related to prosocial behaviour, over time in a sample of 1140 Chinese children from Grades 2 to 5 based on peer nominations and teacher reports. Combining information from the two informants, aggression in Grades 2, 3, and 4 was significantly negatively related to subsequent social competence (peer-assessed sociability, social preference, teacher-rated social competence, and leadership), while this was not the case in Grade 5. In contrast, levels of social competence were not related to aggressive behaviour one year later. These findings hence support the hypothesis of a unidirectional effect from aggression to later social competence, which includes aspects of prosocial behaviour, but not from social competence to aggression. The authors argue that earlier aggressive behaviour may elicit negative social evaluations of others, which may in turn lead to lower levels of social competence and fewer opportunities to develop a healthy self-confidence.

### The current study

In the current study we tested the reciprocal links between prosocial behaviour and aggressive beahviour in a five-year longitudinal study using a large, ethnically diverse urban sample of 1,334 children (aged 7 to 11) from Zürich, Switzerland. In addition, as peer relations emerge as key aspects of both of these behaviours in prior research, we examine peer difficulties as a potential mediating mechanism between the two behaviours. Given that the developmental relations between these two behaviours have not yet been clearly understood, these were first tested independent of peer difficulties as a possible mediating factor in their association. We utilised a large, representative sample of girls and boys and examined the bidirectional cross-lagged links between aggressive and prosocial behaviours based on teacher, parent and child self-reports. This was done because research has shown that the correlations between parent, teacher and child reports are modest, thus suggesting that it is crucial to rely on multiple informant reports when assessing behavioural functioning (Youngstrom et al. 2000). Given the extant research related to sex differences with respect to both aggressive and prosocial behaviours, sex was tested as a potential moderator of the relations between these two behaviours.

Next, given the evidence suggesting that experiences of peer difficulties relate to both, the engagement in aggressive behaviour and prosocial behaviour, we anticipated that such experiences (not perceived as being popular, being victimised, and isolated by peers) will be positively related to aggressive behaviour and negatively to prosocial behaviour. In addition, we expected experiences of peer difficulties to mediate the link between the two behaviours over time.

Consideration has been given to the choice of measure of aggressive behaviour. Several types of aggressive behaviour have been identified (e.g., Murray-Close and Ostrov 2009) both with respect to the “form” of aggressive behaviour (i.e., whether it is expressed physically or in the form of a threat or harm to relationships) and the “function” that it serves (i.e., reactive, or impulse and anger oriented; or proactive, that is goal oriented). In the current study we opted to utilise the broader overt aggressive behaviour scale, which included pro-active, reactive and physical aggression. This broader scale was used for two reasons: First, we wanted to utilise the most robust measure of aggression since this is, to our knowledge, the first study exploring the longitudinal link between these behaviours; second, indirect or relational aggression is more difficult to assess by raters such as teachers or parents as it is often concealed and more difficult to observe (e.g., Kuppens et al. 2013).

We focused on examining these developmental links between ages 7 and 11 as these developmental periods have been identified as key transitional periods from childhood through adolescence to adulthood. During these periods, children experience marked changes in their social lives, which expand beyond their family to include peers and teachers. They develop significant cognitive, emotional and social competencies necessary for later functioning (e.g., Huston and Ripke 2010).

## Method

### Participants

The data were drawn from an ongoing combined longitudinal and intervention study, the Zürich Project on the Social Development of Children and Youth (*z-proso*). The gross sample at the initial assessment consisted of all 1,675 first graders from 56 public elementary schools. Of all approached parents, 81.3% (*n* = 1,361) consented to their child’s participation at wave 1 (W1) and 74% (*n* = 1240) participated in the parent interview at W1. In line with the requirements for ethical conduct in survey-based research with human subjects in Switzerland outlined by the Association of the Swiss Ethics Committee (2009), written informed consent was collected from the parents at the beginning of the study (at W1 valid until W3) and again at the beginning of W4 and from the children from age 13 onwards. Four data collection waves took place between 2004/5 and 2009/10 when the children were 7, 8, 9 and 11 years old (each year through Grades 1 to 3 and Grade 5 corresponding to Ws 1 to 4 across 5 years) and information was collected from parents, teachers and children. Two universal prevention programmes were introduced into the study with the aim to reduce children’s externalising problems. In a factorial design, schools were randomly assigned to a control condition, the Triple-P (Positive Parenting Programme) programme, the social and emotional skills intervention PATHS (Promoting Alternative Thinking Strategies), and a combined (PATHS and Triple P) condition. Findings on the interventions are reported in Malti et al. (2011). In brief, they yielded very limited, if any, evidence of intervention effects. In the present study, we included the two interventions as covariates in all analyses. However, in line with previously reported findings no systematic intervention effects were found.

We analysed data from all three informants from W1 to W4 of data collection. Data were included for all children, teachers and parents, who participated in the first and in at least one subsequent data collection wave resulting in a sample of 1,334 children; 1,191 parents and 1,325 teachers. At W1, the children’s age was *M =* 7.45 years (*SD* = .39). The retention rate from W1 to W2, when the children’s age was *M =* 8.11 (*SD* = .38) was 97% for the child, 95% for the parent, and 96% for the teacher assessments; from W1 to W3 (age *M =* 9.21, *SD* = .37), the retention rate was 96% for the child, 95% for the parent, and 94% for the teacher assessment; and for W1 to W4 (age *M =* 10.70, *SD* = .38), the retention rate was 83% for children, 86% for parent, and 92% for the teacher assessment.

Sample attrition effects were examined by comparing the children at W4 with those who did not participate at W4 (*n* = 275) on demographic variables (i.e., SES and sex) and revealed no significant differences. Of the 1,334 children in the study 51% were boys; at W1 78% lived with both of their biological or adoptive parents, 20% with their biological mother only, and 2% with their biological father only, with foster parents or in residential care (Eisner et al. 2007; Eisner et al. 2011).

Twenty-five percent of the primary caregivers had little or no secondary education, 30% had vocational training, 29% had attended vocational school, had a baccalaureate degree or advanced vocational diploma, and 16% had a university degree. Eleven percent of the children and 46% of both parents were born outside of Switzerland (> than 80 countries). All contact letters and interviews were translated by native speakers into the nine most frequently spoken foreign languages.

### Procedure

At each wave information was collected from the child, the primary caregiver^a^, and the teacher. Computer-assisted 45-minute-long interviews were conducted with the children at school at W1 to W3 and with a parent at W1 to W4 at each child participants’ home. In W4, children completed a written questionnaire. Each child’s teacher completed a questionnaire at all four waves.

### Measures

#### Parent and teacher ratings of aggressive and prosocial behaviour

For the parent and teacher ratings, the Social Behaviour Questionnaire (SBQ; Tremblay et al. 1991) was utilised. The SBQ is a 55-item paper and pencil questionnaire rated on a 5-point Likert scale from never = ‘0’ to very often = ‘5’. It is used to rate children’s psychosocial functioning across ten subscales contributing to five higher order scales. This study utilised two scales of the SBQ: mean scores of the overt Aggressive Behaviour and Prosocial Behaviour scales.

The overt *Aggressive Behaviour scale* included eleven items in total, tapping into pro-active aggression (four items; e.g. ”The child encourages others to pick on a particular child”), reactive aggression (three items; e.g. “The child is aggressive when he/she is contradicted.”), and physical aggression (four items; e.g. “The child kicks, bites and hits”). Cronbach’s alphas ranged from .77 to .81 with mean alpha .79 for parents and from .93 to .94 with mean alpha .93 for teachers.

The *Prosocial Behaviour scale* consisted of ten items and tapped into behaviours related to helping and empathic behaviour, for example “The child helps someone who is hurt” or “The child listens to others’ point of view”; respectively. Cronbach's alphas ranged from .76 to .80 with mean alpha .78 for parents and from .91 to .92 with mean alpha .91 for teachers.

#### Child rating of aggressive and prosocial behaviour

Children completed the *“Tom & Tina” – Adapted Social Behaviour Questionnaire (T & T).* The T&T adaptation was developed by the research team with the purpose of measuring self-reported aggressive and prosocial behaviour amongst primary-school children parallel to the reports of teachers and parents. It is an adapted computer-based multimedia version of the SBQ that consists of a series of 54 drawings displaying specific behaviours of a child called “Tom” or “Tina” based on the child’s sex. For each drawing the child is asked by a voice recorded on the computer whether he/she happens to do what is shown on the drawing and responds by pressing the “Yes” or “No” button at the bottom of each screen. The administration was adapted from the “Dominic Interactif” (Scott et al. 2006) measure with a demonstrated moderate to excellent reliability and validity for young children (Campbell et al. 2006). The computer-based version of the T & T was administered to children at W1 to W3 and its parallel paper and pencil version was administered at W4. We utilised the prosocial and overt aggressive behaviour subscales comprised of parallel items to the SBQ scales described above. Cronbach's alphas ranged from .55 to .62 with mean alpha .60 for prosocial behaviour and from .72 to .79 with mean alpha .76 for aggressive behaviour. The means in Table 1 represent the means for the number of items they responded with “Yes”.

**Table 1:** **Descriptive statistics (means, standard deviations) for aggressive and prosocial behaviour of boys and girls at each wave of measurement by each informant**

		**Boys**		**Girls**					
**Informant**	**Variable (age)**	***M***	***SD***	***M***	***SD***	**min**	**max**	***p***	***d***
Teacher	Prosocial (7)	1.939	.836	2.412	.737	0	4.00	<.001	0.59
	Prosocial (8)	2.033	.792	2.516	.777	0	4.00	<.001	0.62
	Prosocial (9)	2.130	.835	2.673	.739	0	4.00	<.001	0.68
	Prosocial (11)	1.956	.748	2.451	.750	0	4.00	<.001	0.65
Parent	Prosocial (7)	2.478	.543	2.670	.490	0.60	4.00	<.001	0.37
	Prosocial (8)	2.598	.538	2.780	.498	1.00	4.00	<.001	0.35
	Prosocial (9)	2.563	.528	2.774	.512	0.50	4.00	<.001	0.40
	Prosocial (11)	2.587	.555	2.831	.529	0.80	4.00	<.001	0.42
Child	Prosocial (7)	.796	.185	.841	.156	0	1.00	<.001	0.28
	Prosocial (8)	.864	.163	.908	.120	0	1.00	<.001	0.31
	Prosocial (9)	.887	.157	.935	.108	0	1.00	<.001	0.35
	Prosocial (11)	.860	.166	.928	.095	0	1.00	<.001	0.50
Teacher	Aggressive (7)	.721	.764	.448	.555	0	4.00	<.001	0.40
	Aggressive (8)	.645	.682	.457	.571	0	3.45	<.001	0.30
	Aggressive (9)	.678	.686	.452	.560	0	3.55	<.001	0.36
	Aggressive (11)	.678	.774	.384	.535	0	3.75	<.001	0.44
Parent	Aggressive (7)	.669	.445	.539	.381	0	2.75	<.001	0.33
	Aggressive (8)	.721	.457	.605	.415	0	2.58	<.001	0.27
	Aggressive (9)	.701	.436	.595	.415	0	2.50	<.001	0.27
	Aggressive (11)	.562	.380	.453	.340	0	2.41	<.001	0.30
Child	Aggressive (7)	.187	.176	.157	.170	0	1.00	.002	0.18
	Aggressive (8)	.164	.179	.115	.140	0	0.92	<.001	0.30
	Aggressive (9)	.152	.176	.100	.131	0	0.92	<.001	0.33
	Aggressive (11)	.245	.212	.163	.164	0	0.92	<.001	0.43
Teacher	Peer difficulties (7)	1.758	.707	1.710	.715	1	5.00	ns	
	Peer difficulties (8)	1.646	.665	1.633	.651	1	4.67	ns	
	Peer difficulties (9)	1.737	.769	1.684	.708	1	5.00	ns	
	Peer difficulties (11)	1.830	.820	1.778	.777	1	5.00	ns	

Note: *d* – Cohen’s d estimate of effect size.

#### Teacher rating of peer difficulties

At each wave of data collection, teachers answered three questions to rate the degree to which each child is “popular”, “victimised” and/or “isolated” by peers on a 5-point Likert scale from ‘does not apply at all’ to ‘applies very much’. The three items were combined into composite scores, with being popular reverse-scored. The scores for W2 to 4, which yielded Cronbach’s alphas .75, .78, .80, respectively, were utilised in the analyses. This scale was specifically developed for the purposes of this study based on a review of literature related to peer rejection and negative peer experiences. At the time this longitudinal project was launched (in 2004/2005) peer rejection was most commonly measured via peer nomination sociometric tools (Lev-Wiesel et al. 2013). These were deemed not sufficient or feasible for the then six-year old participants of the current study. For consistency of measurement over time, the same measure was utilised during subsequent data collection points.

### Data analytic approach

**Figure 1:**
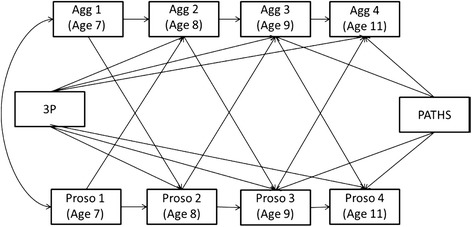
Autoregressive cross-lagged model of the association between prosocial behaviour and aggressive behaviour. Note: Autoregressive pathways are displayed as the pathways within constructs over time (e.g., prosocial behaviour at W1 to prosocial behaviour at W2). Cross-lagged pathways are displayed as the pathways between constructs over time (e.g., prosocial behaviour at W1 to aggressive behaviour at W2). Control variables – exposure to Triple P and/or Paths – were regressed on the relevant waves. Due to its implementation at W2, Triple P was regressed on aggressive and prosocial behaviour at Ws 2, 3, and 4. Due to its implementation one year later at W3, exposure to Paths was regressed only on aggressive and prosocial behaviour at Ws 3 and 4. Not displayed are residual correlations, which were estimated as described in the data analytic plan.

Data analyses were conducted via multiple-group cross-lagged regression models in a structural equation modelling (SEM) framework using the statistical software AMOS (Version 19; Arbuckle 2010; see Figure 1). SEM provides a confirmatory approach to data analysis in which the expected set of structural relations among variables is specified a priori and modelled simultaneously. It also allows for a direct comparison of model parameters across groups (e.g., across boys and girls) through multiple group modelling (Muthén et al. 1997).

First-order autoregressive and cross-lagged pathways of association were simultaneously evaluated. In a first-order autoregressive model, variables are represented as causes of themselves over time. Therefore, autoregressive pathways estimate the association between prosocial behaviour at time t_n_ with prosocial behaviour at time t_n+1_ as well as the association between aggressive behaviour at time t_n_ and aggressive behaviour at time t_n+1_. The autoregressive pathways were allowed to vary across time to allow for the changes in the level of influence that behaviours at time t_n_ have on the same behaviours at time t_n+1_ as children grow older. Aggressive and prosocial behaviours will be modelled in this way as extensive previous literature suggests that past behaviour is often the best predictor of current behaviour (e.g., Crick 1996; Eivers et al. 2012).

Cross-lagged models (e.g., Kenny and Harackiewicz 1979) have been widely used in developmental research to assess bi-directional time-lagged relations (e.g., Defoe et al. 2013). The cross-lagged associations represent relations between prosocial behaviour at time t_n_ and aggressive behaviour at time t_n+1_ as well as the reciprocal association between aggressive behaviour at time t_n_ and prosocial behaviour at time t_n+1_. These effects were allowed to vary across time to examine change in the reciprocal association between aggressive and prosocial behaviour from age 7 to age 11. Concurrent residual correlations between aggressive and prosocial behaviour at the same time of assessment were estimated and allowed to vary over time as were the residuals within construct variances. The intervention conditions (attendance/engagement in the intervention) were included in the models as covariates to account for possible effects on behaviour; Triple P at Ws 2, 3, and 4 as it was implemented when the children were in Grade 2 and Paths at Ws 3 and 4 as it was implemented when the children were in Grade 3. The autoregressive models were set up as multiple-group analyses to examine the association between aggressive and prosocial behaviour by sex. Within this framework, structural models with the associations between aggressive and prosocial behaviour over time were assessed independently in three separate models; one based on each type of informant (child, parent, teacher). The research question of whether sex moderates the associations between aggressive and prosocial behaviour was assessed in each of the models. A series of nested models were fit to the data in which each of the cross-lagged parameters were constrained to be equal across sexes.

**Figure 2:**
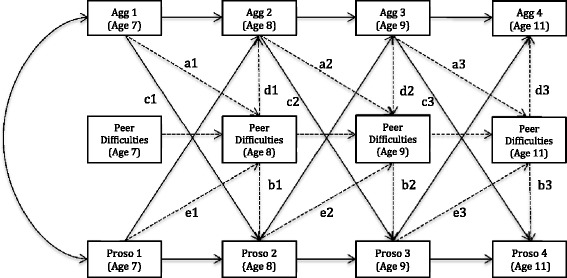
Autoregressive cross-lagged model of the association between prosocial behaviour and aggressive behaviour, including mediation by peer difficulties. Note: Not displayed are pathways controlling for the effects of treatment as pictured in Figure 1 and residual correlations. All of these were estimated as described in the data analytic plan. The dotted lines represent the influences by peer difficulties; paths a1, a2, and a3 represent the influence of aggression at time t on peer difficulties at time t+1; paths b1, b2, and b3 represent the influence of peer difficulties at time t on prosocial behaviour at time t. Paths c1, c2, and c3 represent the direct influence of aggressive behaviour at time t on prosocial behaviour at time t+1.

Finally, nested mediation models were tested to assess the influence of peer difficulties on the association between prosocial and aggressive behaviour at each cross-lag (see Figure 2). Specifically, a model in which the paths from and to peer difficulties were restrained (no mediation) was compared to a model, in which these paths were free to vary (or account for variance). These final models were only tested utilising the teacher reported data for several reasons. First, the reports about peer difficulties were provided by the teachers based on their observation of the children in the classroom, based on which they also rated their engagement in aggressive and prosocial behaviours. Furthermore, some research suggests that teacher reports of behaviour at school are more reliable than those of children (e.g., Ladd and Profilet 1996).

### Missing data

Prior to conducting the final data analysis, missing data patterns were examined. The number of missing values over the four time periods was 4% for teacher reported measures, 8.5% for parent reported measures, and 7% for child reported measures. As missing data for each type of informant were not related to any of the demographic variables collected at W1 (age of parent and child, ethnicity, SES, education, single parenthood), they were handled through the use of Full Information Maximum Likelihood Estimation, which produce valid estimates under the assumption that the missing data are missing at random (MAR; Rubin 1976).

## Results

### Descriptive statistics

Descriptive statistics and paired-samples t-tests revealed significant differences in the mean levels of boys’ versus girls’ aggressive and prosocial behaviours at each wave of data collection for all informants (see Table 1). At each wave, boys rated themselves lower and were rated lower by both their parents and teachers on prosocial behaviour and higher on aggressive behaviour. Effect sizes for sex differences in prosocial behaviours ranged from small to medium, with largest effect sizes observed based on teacher reports. The effect sizes for differences in aggressive behaviour remained in the small range (maximum 0.44 according to teacher reported aggression at age 11; see also Nivette et al. 2014). There were, however, no significant mean differences in the rate of peer difficulties experienced by boys and girls as reported by their teachers.

The inter-correlations between the study variables are displayed in Table 2. The within-informant correlations between the ratings of the child’s aggressive and prosocial behaviour were small to medium, but negative and significant at each wave. The correlations between teacher reported aggressive behavior and peer difficulties were positive and medium in size. On the other hand, the correlations between prosocial behaviours and peer difficulties were negative and small.

**Table 2:** **Zero-order correlations between variables in the study at each wave of data collection**

	**Aggressive and prosocial behaviour**			**Aggressive behaviour and peer difficulties**	**Prosocial behaviour and peer difficulties**
	**Teacher**	**Parent**	**Child**	**Teacher**	**Teacher**
Age (7)	-.30**	-.24**	-.08*	.45**	-.37**
Age (8)	-.30**	-.25**	-.23**	.48**	-.31**
Age (9)	-.33**	-.23**	-.23**	.38**	-.34**
Age (11)	-.36**	-.35**	-.22**	.34**	-.33**

Note: ** *p* < .01; * *p* < .05 (2-tailed).

### Structural equation models

All SEM models were evaluated using recommended fit indices, including root mean square error of approximation (RMSEA), where values < .08 indicate acceptable fit and values < .05 indicate good fit; confirmatory fit index (CFI) and normed fit index (NFI) where estimates > .90 indicate acceptable fit and values > .95 indicate good fit (McDonald and Ho 2002). Because the *χ*^*2*^ becomes increasingly sensitive with growing sample size (Marsh et al. 1988), it was not considered for evaluation of model fit. Instead, we used practical fit indexes to test for sex invariance. According to Little (1997), model invariance can be assumed (a) if the overall model fit is acceptable, as indicated by relative fit indexes (e.g., if the CFI is approximately .90 or greater; Marsh et al., 1988; and if the RMSEA is less than .05; Browne and Cudeck 1993); (b) if the difference in model fit is negligible (e.g., ≤.05 for the fit indices) after introduction of the equality constraints; and (c) if the justification for the accepted model is substantively more meaningful and the interpretation is more parsimonious than the alternative model. In addition, we followed recommendations by MacCallum et al. (1996) and used the 95% confidence interval (CI) around the RMSEA to evaluate model fit and for nested model comparisons. Specifically, if the upper bound of the CI is equal to or lower than .05, a close fit of the model to the data can be assumed. Moreover, if the CIs of subsequent nested models overlap with those of preceding, less constrained models, the more parsimonious model is deemed acceptable.

### Sex Invariance

In the first step of the analyses, we examined whether invariance across boys and girls can be assumed. Model invariance across the sexes was assumed to be more parsimonious and was tested in the model for each type of informant by comparing the fit indices of nested models: A model where all the regression weights were free to vary across boys and girls, and a model in which these regression weights were constrained to be equal (see Table 3). Comparison of fit indices supported sex invariance (no significant sex differences) in the predicted paths between aggressive and prosocial behaviour over four points of data collection, from 7 to 10 years of age. This was the case for each of the models (see Table 3); teacher reported (NFI = .939, CFI = .940, RMSEA = .050), parent reported (NFI = .922, CFI = .921, RMSEA = .060) and child self-reported (NFI = .907, CFI = .904, RMSEA = .038). Chi-square difference tests were also carried out for each informant and provided further support for sex invariance (critical value 12.59 > observed difference of 5.89, 4.66 and 10.74 for the teacher, parent and child model, respectively; *p* < .05). Given support for sex invariance in the fit of each of the models, individual paths are interpreted for the constrained models (the sample overall) and not separately for boys and girls.^b^

**Table 3:** **Summary of nested model tests regarding sex invariance**

		**NFI**	**CFI**	**RMSEA**	**CI 95% RMSEA**	**Δdf**	***χ*** ^***2***^	**df**
Teacher	Unconstrained	.931	.931	.059	.052-.065		256.040	38
	Constrained (Invariant)	.939	.940	.050	.044-.055	6	261.930	44
Parent	Unconstrained	.919	.918	.074	.067-.081		386.755	38
	Constrained (Invariant)	.922	.921	.060	.055-.065	6	391.420	44
Child	Unconstrained	.923	.919	.044	.037-.051		161.762	38
	Constrained (Invariant)	.907	.904	.038	.033-.044	6	172.507	44

Note: IFI = incremental fit index; CFI = comparative fit index; RMSEA = root mean square of approximation; Δdf = change degrees of freedom.

### Autoregressive relations of aggressive and prosocial behaviours

As expected, prosocial and aggressive behaviour at time t was significantly related to aggressive and prosocial behaviour at t_n+1_, respectively. Previous behaviour significantly predicted the same future behaviour consistently across all waves and all types of informants. This was the case with respect to both aggressive and prosocial behaviour (all *p*s < .001; *B = .*37 to .71 and .27 to .64, respectively).

### Cross-lagged relations between prosocial behaviour and aggressive behaviour

Next, we examined the cross-lagged effects between aggressive and prosocial behaviour. Based on both the teacher- and parent-reported models, increases in aggressive behaviour at time t_n_ consistently and significantly predicted decreases in prosocial behaviour at time t_n+1_ across each of the waves. However, based on both the teacher- and parent-reported models increases in prosocial behaviour at time t_n_ did not predict decreases in aggressive behaviours at time t_n+1_. A similar pattern of negative paths from aggressive behaviour to prosocial behaviour was observed in the model based on children’s self-reports. However, only the paths from aggressive behaviour at age 7 to prosocial behaviour at age 8 reached statistical significance (see Table 4 for all model coefficients).

**Table 4:** **Cross-lagged and autoregressive unstandardised estimates of aggressive and prosocial behaviour, and treatment effects**

	**Teacher**	**Parent**	**Child**
	***B***	***B***	***B***
*Cross-lagged*			
Aggressive (7) → Prosocial (8)	-.053*	-.149***	-.079***
Aggrresive (8) → Prosocial (9)	-.102***	-.089***	-.034^¥^
Aggressive (9) → Prosocial (11)	-.092**	-.073*	-.014
Prosocial (7) → Aggressive (8)	-.025	-.028	-.018
Prosocial (8) → Aggressive (9)	-.033	-.025	.012
Prosocial (9) → Aggressive (11)	-.015	-.030	.039
*Autoregressive*			
Aggressive (7) → Aggressive (8)	.633***	.713***	.371***
Aggressive (8) → Aggressive (9)	.624***	.686***	.469***
Aggressive (9) → Aggressive (11)	.377***	.557***	.453***
Prososcial (7) → Prosocial (8)	.598***	.575***	.269***
Prosocial (8) → Prosocial (9)	.617***	.641***	.371***
Prosocial (9) → Prosocial (11)	.265***	.618***	.291***
*Triple P*			
Aggressive (8)	.060*	.001	-.014^¥^
Aggressive (9)	.001	-.001	-.006
Aggressive (11)	-.038	.008	-.014
Prosocial (8)	.063^¥^	-.006	.002
Prosocial (9)	-.159***	-.001	-.008
Prosocial (11)	-.022	.026	.002
*Paths*			
Aggressive (9)	.029	-.010	.006
Aggressive (11)	-.027	-.013	-.009
Prosocial (9)	-.019	-.031	-.015*
Prosocial (11)	-.053	.048^¥^	.003

Note: The numbers in brackets indicate age at time of measurement. The presented coefficients are ustandardised estimates recommended by Kline (1998) to be used when reporting results in AMOS, as only those (and not the standardised estimates) are affected by identification constraints (Arbuckle, 1995).

***p < .001, **p < .01; *p < .05; ^¥^ < .10.

### Mediation by peer difficulties

Next we tested a model (see Figure 2), in which peer difficulties at t_n+1_ was included as a mediator of the links between aggressive and prosocial behaviours one year later (at t_n+1_). This model yielded a significant goodness of fit for the overall model, *χ*^*2*^ (126) = 386.907; *p* < .001, however, it also showed adequate approximate fit indices (NFI = .927; CFI = .946; RMSEA = .038). To further assess the fit of the mediation model, we tested it against the original model. Specifically, a model, in which the paths from (b1-b3; d1-d3; see Figure 2 dotted lines) and to peer difficulties (a1-a3; e1-e3; see Figure 2 dotted lines) were free (free to mediate), was compared to a model, in which these paths were restrained. The comparison of the two models yielded a significant chi-square difference score; *χ*^*2*^_*diff*_*(14)* = 1504.999, *p* = .001. Thus, we deemed the mediation model to be a better fit and appropriate for interpretation.

The interpretation of the individual paths suggested that the significant links from aggressive behaviour to prosocial behaviour one year later were mediated by the influence of peer difficulties. Specifically, in the model (Figure 2) where peer difficulties were free to be estimated as predicted by previous levels of aggressive behaviour (paths a1, a2, and a3) and predicting concurrent levels of prosocial behaviour (paths b1, b2, and b3) the direct links between aggressive and prosocial behaviour one year later (paths c1, c2, and c3) were no longer significant (see Table 5; the right most column in the table corresponds to the paths in Figure 2). Instead, aggressive behaviour significantly predicted lower levels of peer difficulties one year later at ages 8 and 9 but not at age 11 (*B*^*c*^ *=* .292; .084 and .057; a paths respectively). Furthermore, peer difficulties at age 8, 9 and 11 were a significant predictor of both aggressive behaviour and prosocial behaviour concurrently (each at *p* < .001). Specifically, they predicted higher levels of aggression at each age (*B =* .283; .163 and .232; d paths respectively) and lower levels of prosociality (*B = −*.216; −.208 and -.284; b paths respectively). Thus, given that aggressive behaviour predicted peer difficulties, which in turn predicted prosocial behaviour, aggressive behaviour seems to be mediated or exert influence on prosocial behaviour through its influence on peer difficulties. Higher levels of peer difficulties as a result of previous aggressive behaviour appear to be the mechanism through which aggressive behaviour is related to lower levels of prosocial behaviour later on. Interestingly, however, prosocial behaviour predicted a lower level of peer difficulties only from age 7 to age 8 (*B = −*.152) but not at later ages.

**Table 5:** **Cross-lagged and autoregressive unstandardised estimates of aggressive and prosocial behaviour, and peer difficulties**

	**Constrained model**	**Peer mediation model**	
	***B***	***B***	**Paths**
Aggressive (7) → Prosocial (8)	-.053*	.010	c1
Aggressive (8) → Prosocial (9)	-.102***	-.027	c2
Aggressive (9) → Prosocial (11)	-.092**	-.039	c3
Prosocial (7) → Aggressive (8)	-.025	.017	
Prosocial (8) → Aggressive (9)	-.033	-.010	
Prosocial (9) → Aggressive (11)	-.015	.002	
Aggressive (7) → Peer difficulties (8)		.292***	a1
Aggressive (8) → Peer difficulties (9)		.084**	a2
Aggressive (9) → Peer difficulties (11)		.057	a3
Prosocial (7) → Peer difficulties (8)		-.152***	e1
Prosocial (8) → Peer difficulties (9)		-.034	e2
Prosocial (9) → Peer difficulties (11)		.027	e3
Peer difficulties (8) → Aggressive (8)		.283***	d1
Peer difficulties (9) → Aggressive (9)		.163***	d2
Peer difficulties (11) → Aggressive (11)		.232***	d3
Peer difficulties (8) → Prosocial (8)		-.216***	b1
Peer difficulties (9) → Prosocial (9)		-.208***	b2
Peer difficulties (11) → Prosocial (11)		-.284***	b3

Note: The right most column corresponds to the pathways in Figure 2. Pathways for which estimates are not presented were constrained in the constrained model.

***p < .001, **p < .01; *p < .05.

## Discussion

Both, aggressive behaviour and prosocial behaviour, have been identified as crucial in children’s social development (Eisenberg 2000; Eisenberg et al., 2015; Eisner and Malti 2015). While both behaviours have been studied extensively independently, less is known about the way they relate to each other throughout development. The current study contributed to this understanding by examining the bidirectional cross-lagged links between aggressive and prosocial behaviours in a large-scale sample of boys and girls from age 7 to 11. The relations were examined on the basis of teacher, parent and child self-reports.

### Aggressive behaviour and prosocial behaviour

Our first main finding was that both, aggressive behaviour and prosocial behaviour one year prior, were strong predictors of the same behaviour one year later, thus suggesting considerable stability in both behaviours. There is evidence in support of stability of aggressive behaviour across normative and high-risk samples from early childhood (Crick et al. 2006) through adolescence (Piquero et al. 2012). Much less is known about the stability or change in prosocial behaviour over time (Hay and Cook 2007). The handful of studies which have explored these trends suggest a modest continuity in prosocial behaviours according to teacher reports, but not peer nominations measured at two time points, from age 9 to 12 (Zimmer-Gembeck et al. 2005) and from age 5 to 6 (Eivers et al. 2010). The current study provides support for the continuity of both aggressive and prosocial behaviours by demonstrating these relations across four time points, from age 7 to 11 in a large sample. Importantly, the level of stability was similarly high for prosocial behaviour as it was for aggressive behaviour.

We also found evidence for the one-directional prediction of aggressive behaviour on prosocial behaviour one year later but not vice versa. Children’s elevated levels of aggressive behaviour at time t_n_ predicted a decreased level of their engagement in prosocial behaviour at t_n+1_ after controlling for their propensity to engage in prosocial behaviour at t_n._ In contrast, no evidence in support of the effects in the opposite direction was found. Our results suggest that this pattern of findings holds equally for boys and girls and was evident in the parent and the teacher reports. Findings for the child reports were in the same direction, but were not significant, with the exception of the effects of aggressive behaviour at t_1_ predicting decreased prosocial behaviour at t_2_. The lower consistency in the results for the child self-reports can be due to the fact that the child data have lower reliability, resulting in attenuated observed measures of existing relationships. Nevertheless, it is important to note that teacher, parent and child reports were all positively correlated across all time points with respect to both types of behaviours. Given that the pattern of findings is consistent across informants, we believe that our findings provide evidence of the one-directional pattern of effects of levels of aggressive behaviour on levels of prosocial behaviour but not vice versa.

Taken together, these findings are consistent with the findings of Chen et al. (2010) based on a similar design in a similar sample of children in China. The authors found that aggressive behaviour at t_n_ was related to social competence at t_n+1_, but that social competence did not predict later aggressive behaviour. Although social competence as measured by Chen and colleagues and prosocial behaviour as measured in this study are not the same construct, they are closely related. The consistency of findings in two different cultures suggests that they may reflect universal rather than culturally specific dynamics.

Conceptually, there are several possibilities of how one type of behaviour can influence subsequent behaviour patterns within the same individual. In this paper we expanded previous research by examining the role of peers and specifically peer difficulties on facilitating this link. Our findings suggest that aggressive behaviour is related to children’s subsequent experiences of peer difficulties, which in turn is related to decreases in prosocial behaviours. These findings are consistent with a transactional model developed by Sameroff (2000), which proposes that an individual’s behaviour has effects on the social environment, which in turn triggers change in another behaviour domain. In other words, our findings suggest that children who engage in aggressive behaviour may elicit negative social evaluations by others, which are associated with peer difficulties and in turn may lead to fewer opportunities to practice and further develop social competencies. However, as prosocial behaviour and peer difficulties are measured at the same time, it is also possible that increases in aggression lead to decreases in prosocial behaviour, and this in turn results in increases in peer difficulties. This possibility warrants further examination.

In line with our findings and our proposed primary interpretation, some research suggests that children’s social reputation among peers significantly decreases when they continuously behave overtly aggressively (e.g., Card et al. 2008). These children are often rejected by prosocial peers and continue to be rejected by peers overall even one year later (Lansford et al. 2010). Also, aggressive children may not readily express moral emotions based on respect, reciprocity and cooperation, and hence lower the readiness of more socially competent children to engage in interactions with them (Gasser and Malti 2012). Thus, aggressive behaviour is likely to be linked to peer difficulties because victims of aggressive behaviour may avoid subsequent contact with the aggressors due to a fear of further victimisation (Rubin et al. 2009). Some research suggests that based on their experiences of difficulties with prosocial peers and acceptance by aggressive peers, children develop negative views of themselves (Rudolph and Clark 2001), which may lead to lowered motivation to act in a prosocial way. Others (e.g., Volk et al. 2012) suggest that aggressive behaviour in children and adolescents has strategic and evolutionary roots. Following this argument, children who successfully aggress against others may have fewer incentives to engage in cooperative behaviour.

Notably, peer difficulties were a significant mediator between aggressive behaviour and prosocial behaviour up until age 9. However, it was no longer significant in linking aggressive behaviour at age 9 to prosocial behaviour at age 11. Possibly, aggressive behaviour in younger children exerts a greater influence on future peer difficulties than in older children, where the pattern of peer difficulties may already be set, aggression becomes more valued (or less disliked) and/or children transfer into different classrooms/schools as it was the case in this study. Both of these hypotheses warrant further inquiry to further elucidate the role of peer difficulties in the development of these behaviours from childhood to pre-adolescence. The current study utilised a new measure of peer difficulties and as such these findings are not directly comparable with other studies exploring the role of peer rejection and victimisation specifically.

Future research needs to extend our study and investigate the moderating and mediating role of various other dimensions of peer relationships (e.g., friendship quality, characteristics of friends and peers, etc.) and other processes, unexplored in the current study, that may also contribute to the link between aggressive and prosocial behaviour. For example, there is ample evidence suggesting that aggressive children tend to develop friendships with other aggressive children (e.g., Bowker et al. 2007). Children who are surrounded by aggressive peers may also be under peer pressure and at first opt to not engage in prosocial behaviours so as to appear tough, avoid ridicule, or feel accepted as part of the peer group (e.g., Pepler et al. 2008). Through these associations, children may be exposed to fewer opportunities to practice previously acquired, or to acquire new, social skills, which would allow them to engage in more prosocial behaviours.

Each of the above explanations adopts the more common interpretation in linking higher levels of aggression to decreased levels of prosocial behaviour later. However, the opposite is possible as well. Specifically, it is plausible that children’s low levels of aggressive behaviour predicted an increased level of their engagement in prosocial behaviour later after controlling for their propensity to engage in prosocial behaviour. In the current study we did not examine the specificity of these links, but this question represents another important future direction for research in this area.

Interestingly, prosocial behaviour in the previous year did not negatively predict aggressive behaviour in the following year according to any of the informants. In other words, engaging in more prosocial behaviour in one year did not predict decreases in aggressive behaviour the next year. This may imply that children’s engagement in more helpful and considerate behaviours is not directly linked to their engagement in less aggressive behaviours. Given the low level of aggressive behaviour overall among the children in this sample, it is possible that children with relatively high levels of prosocial behaviour do not engage or engage in only low levels of aggressive behaviour. However, this would not explain why increases in aggressive behaviour one year would predict decreases in prosocial behaviour the next year. Variation in aggressive behaviour over and above the individual propensity might be driven by factors other than other-oriented, prosocial behaviour, for example emotion recognition, empathy, and emotion regulation. Here we did not examine various additional other-oriented social-emotional skills, such as identifying and managing emotions, understanding others’ emotions, and how they may be related to both types of behaviours, and cross-lagged relations on each other over time (see Fraser et al. 2005). Given the importance that is placed on the development of other-oriented, prosocial skills with the goal to decrease aggressive behaviours and increase prosocial behaviours, the examination of these links is a crucial next step in understanding the processes through which positive behavioural outcomes are expected to occur.

Consistent with past research, our results also revealed sex differences in the mean levels of aggressive and prosocial behaviour (Ostrov and Keating 2004). However, the developmental relations between aggressive behaviour and prosocial behaviour were not dependent on the sex of the child. This finding provides further evidence suggesting that the processes through which these two behaviours are related may not be gender-specific. Furthermore, our results suggested that boys and girls experienced similar levels of peer difficulties from age 7 to 11. Similarly, the effects of peer difficulties did not differ in linking aggressive and prosocial behaviours in boys versus girls. These findings are consistent with reports from previous studies (e.g., Crick and Dodge 1996), which found no sex differences in the links between peer rejection and reactive and proactive aggression. Further supporting previous findings but extending research by documenting these links over a five-year period, peer difficulties were consistently concurrently related to increased aggressive behaviours and decreased prosocial behaviour. Thus, this finding further documents the harmful effects of peer difficulties on child development across two behavioural domains.

While the multi-informant measurement of prosocial and aggressive behaviour in a large sample of children over five years constitutes a strength of this study, several limitations should be noted. First, we focused only on direct/overt aggressive behaviour and overt prosocial behaviour, which included helping, sharing, and comforting behaviours. As we pointed out earlier, this approach has its advantages. However, recent evidence suggests that different types of aggressive behaviour, such as relational and physical aggression, may have different developmental links with later prosocial behaviour (Carlo et al. 2003). In the present study, we focused on overt direct aggression, amongst others because indirect aggression is much more difficult to assess by raters such as teachers or parents. Future research is needed to examine the developmental causal pathways between sub-domains of aggressive and prosocial behaviour. For example, future studies should examine the pattern of these relations with respect to relational aggression as it is possible that these will differ for girls versus boys. Furthermore, the aggression and prosocial variables in this study were skewed, as is to be expected in a normative sample. This could have influenced the estimates, however, according to Satorra (2001) non-normality in structural equation models is not a problem with large samples (over 1000 as is the case in this study) and results are robust.

Second, our assessment of peer difficulties was based on teacher reports. While peer difficulties are most commonly observed in the school context and teachers provide solid ratings of peer difficulties, future studies that combine teacher reports and peer nominations may elucidate similarities and differences of these ratings in relation to aggression and prosocial behaviour. Third, while the parent and teacher scales of aggressive and prosocial behaviour were directly adapted from a well-established instrument (SBQ; Tremblay et al. 1991), the parallel child measure involved a greater adaptation due to its computer-administration and dichotomous response style. These adaptations were implemented in order to provide children a more accessible response alternative. The internal reliability of the child scales was relatively low, in particular with respect to prosocial behaviour, hence the results related to the child-reported behaviours should be interpreted with caution and warrant replication.

## Conclusions

Despite some limitations, the current study offers insights into the effects aggressive and prosocial behaviours have on each other with a one to two year lag, and has as such implications for the design of interventions that aim to reduce aggression. Specifically, our findings highlight that prosocial behaviour may not necessarily be seen as a main proximal target of intervention strategies. Our study provides further support for the role of peer difficulties as an important mechanism linking aggressive behaviour and subsequent decreases in prosocial behaviour. Together these findings suggest that promoting positive peer relationship may be an important component of interventions with young people exhibiting behaviour problems.

## Endnotes

^a^From here on we will use the term ‘parent’ to refer to the primary caregiver. The vast majority of primary caregivers (97%) were biological mothers.

^b^Model invariance by immigration status (yes/no) was tested and compared to the unconstrained model was not significantly different than the constrained (invariant) model, suggesting that immigration status did not make a difference in model fit.

^c^The presented coefficients are unstandardised estimates recommended by Kline (1998) to be used when reporting results in AMOS, as only those (and not the standardised estimates) are influenced by identification constraints.
